# Cervical radiculopathy for neurologists: the role of electrodiagnosis

**DOI:** 10.1055/s-0045-1812893

**Published:** 2025-11-28

**Authors:** Lucas Immich Gonçalves, Pedro Helder de Oliveira Junior, José Pedro Soares Baima

**Affiliations:** 1 University Health Network, University of Toronto, Ellen and Martin Prosserman Centre for Neuromuscular Diseases, Toronto ON, Canada.; 2 Pontifícia Universidade Católica do Rio Grande do Sul, Hospital São Lucas, Departamento de Neurologia, Porto Alegre RS, Brazil.; 3 Universidade Federal do Ceará, Hospital Universitário Walter Cantídio, Unidade de Neurofisiologia, Fortaleza CE, Brazil.

**Keywords:** Electromyography, Nerve Conduction Studies, Radiculopathy

## Abstract

Cervical radiculopathy (CR) is a common condition encountered in the general population, usually related to a musculoskeletal degenerative condition. Conventional electroneuromyography (ENMG) consists of nerve conduction studies (NCS) and needle electromyography (EMG), and it is regarded as the most specific diagnostic evaluation in this scenario. Although CR is commonly encountered in clinical practice, ENMG as a diagnostic tool is not often discussed in neurology residency programs. Electromyography has demonstrated modest sensitivity (50–71%) but excellent specificity (approaching 100%) for the diagnosis of CR. It can also provide valuable information about lesion chronicity. In EMG, acute lesions typically present with denervation potentials and reduced recruitment, but with preserved motor unit action potential (MUAP) morphology. In contrast, chronic lesions are characterized by remodeling, with MUAPs showing increased duration, amplitude, and number of phases, in addition to reduced recruitment. The present review aims to provide an overview of the roles of NCS and EMG, while also introducing key terminology commonly encountered in the interpretation of these diagnostic modalities.

**TEACHING POINTS**

1. Electrodiagnostic testing, specifically needle electromyography (EMG), has a high specificity for radiculopathy.
2. An adequate EMG evaluation involves at least 6 muscles, with one being the paraspinal
3. Chronicity in cervical radiculopathy can be estimated based on EMG findings.
4. The presence of fibrillation potentials and positive sharp waves on EMG indicates active denervation and ongoing axonal damage.

## CLINICAL VIGNETTE

**Figure 1:**
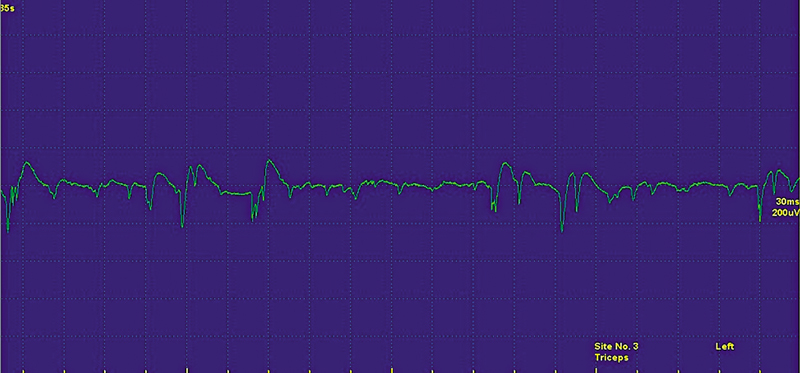
Display showing fibrillations and positive sharp waves in triceps during needle examination.

**Figure 2:**
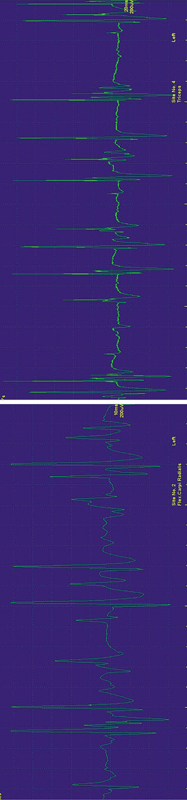
To fully visualize, rotate right the figure. Voluntary activity during needle examination of the left flexor carpi radialis (left) and triceps (right). The MUAP has normal duration and amplitude, but recruitment is reduced. The reduced recruitment is shown as intervals with out potential in the interference pattern. Normally, thebscreen would be full of potentials during this degree of contraction.

A 42-year-old man presented with neck pain radiating down the left arm for 2 months. He reported a bothersome tingling over the middle finger. Physical examination revealed mild hypoesthesia in the left middle finger and reduced triceps reflex. Strength testing was unremarkable. Nerve conduction studies (NCS) were normal. Needle electromyography (EMG) showed signs of active denervation (fibrillations and acute sharp-wave potentials) in the left triceps and flexor carpi radialis muscles ( Figure 1 ). Motor unit action potentials (MUAPs) had normal duration and amplitude, but recruitment was reduced ( Figure 2 ). The ENMG study report was disclosed as a preganglionic motor lesion affecting the left C7 myotome in keeping with a subacute radiculopathy. Magnetic resonance imaging (MRI) of the cervical spine revealed spondylosis with left C6 to C7 foraminal encroachment.

## FROM PRESENTATION TO RESOLUTION: LESSONS LEARNED

Cervical radiculopathy (CR) is a common musculoskeletal degenerative condition encountered in clinical practice, being more frequent in men with a peak between 50 and 60 years old. 1 Population-based studies indicate that CR has an annual incidence of up to 83.2 cases per 100,000 individuals and constitutes one of the leading causes for visits to physicians' offices. 2 3 Symptoms of radiculopathy result from compression of nerve roots or of the spinal nerve ( Table 1 ), and can have different presentations, including pain, sensory loss, weakness, decreased deep tendon reflexes, and a combination of these findings. 1 Despite its frequency, the diagnostic evaluation of radiculopathies with electroneuromyography (ENMG) is often underappreciated in neurology residency programs. This review intends to address this gap.

**Table 1:** Main causes of cervical radiculopathy

Degenerative cervical spondylosis
Disc herniation
Trauma (root avulsion)
Other compressive causes (tumor)
Inflammatory/immune-mediated (vasculitis, Guillain-Barré, paraneoplastic)
Infectious (herpes zoster, Lyme disease)

### Anatomical considerations

There are eight cervical roots and seven spinal vertebrae. This implies that roots from C1 to C7 originate above the corresponding vertebrae, and from T1 downward they originate below the corresponding vertebrae. 3

The upper limb muscles are innervated by axons from C5 to T1 spinal nerves. The motor and sensory nerve roots leave the spinal cord and fuse together at the intervertebral foramen level to become a spinal nerve. This spinal nerve then splits into ventral and dorsal rami as they exit the foramen. The ventral rami will then combine to form the brachial plexus. 4 It is important to highlight that even though muscles are usually innervated by one peripheral nerve, this nerve will carry motor fibers from at least two different roots. 4 This is an important concept to keep in mind, as it justifies why an isolated radiculopathy is not usually implied as a cause of total muscle paralysis and is also important to understand NCS findings and the rationale behind the use of EMG for localization of the probable affected root. 5

In contrast to lumbar radiculopathy, where the most frequent cause is nucleus pulposus herniation, the main reason for CR is decreased disc height along with vertebral degeneration, resulting in foraminal encroachment, responsible for around 75% of cases. 1 3 The remaining cases (20–25%) are due to disc herniation. 1

### Clinical examination

Spinal nerve myotome is the group of muscles innervated by one motor root. Familiarity with myotomal patterns is essential for neurologists, as certain conditions—such as radiculopathies—present with deficits that follow it, instead of peripheral nerve distributions, as in mononeuropathies, plexopathies, or multifocal motor neuropathy. Clinical examination of patients with CR demonstrated that diminished tendon reflexes have the highest specificity and sensory testing has the lowest, when compared with ENMG. 6

Although there is a debate among the best myotomal charts, 7 we consider the following table of muscles and reflexes to be helpful in assessing each individual myotome in a bedside neurological examination ( Table 2 ). 4

**Table 2:** Anatomical considerations during bedside physical examination in a patient with suspected CR

Root	Nerve	Muscle	Main function	Reflex
C4-C5	Dorsal scapular nerve	Rhomboid	Retraction of the scapula with associated adduction of the arm	Scapulohumeral reflex
C5	Supraescapular	Infraspinatus	Shoulder external rotation	Deltoid reflex
	Axillary	Deltoid	Arm abduction (30–90°)	
C6	Musculocutaneous	Biceps brachii	Elbow flexion	Biceps reflex
	Radial	Brachioradialis	Elbow flexion with the forearm partially supinated	Brachioradialis reflex
C7	Radial	Triceps	Elbow extension	Triceps reflex
	Median	Flexor carpi radialis	Wrist flexion	
C8–T1	Ulnar	First dorsal interosseous	Second finger abduction	Finger flexor reflex
	Median	Flexor pollicis longus	Distal thumb flexion	

Abbreviation: CR, cervical radiculopathy.

Note: An adequate needle EMG examination should include at least one muscle of each root (not necessarily these ones) and a paraspinal muscle.

### Is cervical spine imaging sufficient for diagnosis, or is ENMG also necessary for evaluating CR?

The standard ENMG for radiculopathy evaluation encompasses NCS and EMG. Across different studies, the sensitivity of EMG for diagnosing CR ranges from 50 to 71%, often described as a modest sensitivity, but with excellent specificity, with a 100% marging. 5 This contrasts with MRI, as it has a high sensitivity.

One may ask whether both examinations are necessary. A short answer is that both examinations should be performed, since they complement each other.

First, ENMG helps in the differential diagnosis of symptom origin, aids in the localization of myotomal compromise, and grades severity of radiculopathy. The MRI scan identifies a series of abnormalities, not always with clinical relevance. 8 A series of studies demonstrated that EMG-identified radiculopathies have better surgical outcomes. 8

### Contribution of NCS to radiculopathy evaluation

We highlight the importance of NCS to rule out differential diagnoses, as well as diagnose comorbid conditions, such as peripheral neuropathies. Often, patients' complaints will not be clearly indicative of CR, and physical examination findings can be misleading because of low sensitivity.

The dorsal root ganglion is located outside the central nervous system, between the sensory root and the spinal nerve. 4 As a result, isolated compression at the root level does not damage the ganglion, and the distal sensory fibers do not undergo degeneration. Since sensory NCS evaluate the distal aspect of the nerve up to the sensory ganglia, they are typically normal. This finding is named preganglionic.

In motor NCS, we may find abnormalities like decreased amplitudes due to axonal loss in severe CR. Routine NCS in the upper limbs evaluate mainly C8-T1 roots, since registers are usually in distal muscles in the hand innervated by median and ulnar nerves. Consequently, abnormalities aren't expected in NCS for CR affecting C5 to C7 levels, but subtle motor abnormalities may be found in severe C8 to T1 radiculopathies. 9 Radiculopathies at these levels are less frequent than others, and motor NCS are frequently unremarkable.

Some conditions may present comorbidly or require exclusion through specific tests. Radiculopathies may coexist with mononeuropathies, such as carpal tunnel syndrome. In the differential diagnosis of C8 to T1 radiculopathy, ulnar neuropathy at the elbow and true neurogenic thoracic outlet syndrome must also be considered. These conditions can present with overlapping symptoms, but NCS can accurately distinguish them. Comprehensive protocol with adequate diagnosis can drastically change patient management. 10 Long-latency responses, such as the H-reflex, can be useful in detecting proximal nerve root involvement. In the upper limbs, the H-reflex may aid in the evaluation of C7 radiculopathy, with one study demonstrating a sensitivity comparable to that of EMG. 11

A special situation occurs with suspected radiculopathy in a patient with neuropathy. Sometimes, neuropathy is so severe that it precludes the evaluation of radiculopathy with EMG. To overcome this issue, the neurophysiologist can sample a more proximal muscle that is not overshadowed by neuropathy, then compare the findings with the contralateral side.

### How can a neurologist determine whether an EMG/NCS study followed the adequate protocol for CR evaluation?

To improve identification and correctly display the level of radiculopathy, Dillingham et al. determined sensitivity and specificity in a series of muscle combinations. The recommendation derived from their work is to sample at least six muscles of different myotomes, one of them being a paraspinal muscle. 5

Recommendations have also been proposed for defining an abnormal examination: a diagnosis of radiculopathy is supported when at least two muscles innervated by the same root, but supplied by different peripheral nerves, show neurogenic abnormalities that cannot be attributed to another cause ( Table 3 ). 5 Based on these recommendations, neurologists can recognize an adequate examination protocol when there is a sufficient number of muscles studied and at least two abnormal muscles innervated by different nerves.

**Table 3:** Simplified findings in needle EMG study of radiculopathies

Time course	Recruitment	Active denervation	Motor unit morphology
Acute	Reduced	Absent	Normal
Subacute	Reduced	Present	Normal
Chronic with activity	Reduced	Present	⇑ Amplitude and duration
Chronic without activity	Reduced	Absent	⇑ Amplitude and duration

Abbreviation: EMG, electromyography.

### How should ENMG findings be interpreted in the context of CR?

The ENMG report may be difficult even for neurologists to understand, which may be related to several expressions used across different laboratories due to lack of uniformity in test reports, adding further complexity to this issue. 12 We will briefly review some major findings to improve understanding of the report.

### Activity

From the onset of the pain, signs of denervation are identifiable after around 14 to 21 days. In the acute setting, this is often the best time to perform EMG studies, since fibrillations and positive sharp waves are better indicators than reduced recruitment, especially if they are found in distal, proximal, and paravertebral muscles. 5 8 12

Active denervation can persist for several years, most commonly in distal muscles. 13 Chronic lesions, however, may also be associated with episodes of pain exacerbation. When fibrillation potentials are detected on EMG in this scenario, they are interpreted as signs of ongoing activity or active denervation ( Figure 1 ). 12

### Chronicity

**Figure 3:**
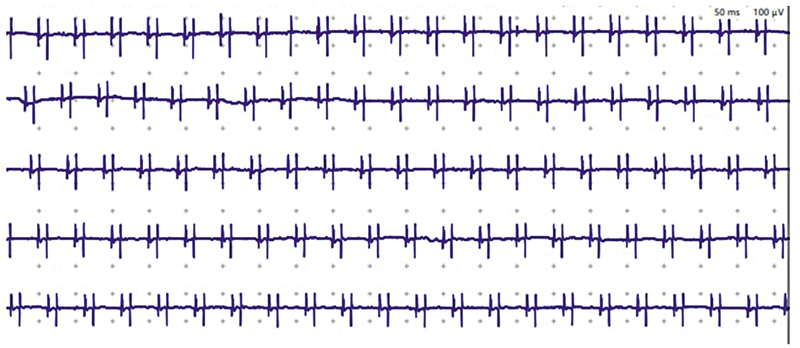
Complex repetitive discharge, a hallmark of chronicity in radiculopathy. These are perfectly regular spontaneous discharges, with identical morphology noted as a machine-like sound on EMG with an abrupt onset and termination.

Radiculopathy is described as acute, subacute, or chronic. This is done mainly by reviewing the characteristics of MUAP. Acute radiculopathy has not been reinnervated, therefore, these units remain in their usual amplitude and duration. The neurophysiologist can identify reduced recruitment by observing a failure to appropriately increase the MUAP number with increasing voluntary muscle contraction, as would normally be expected. Subacute radiculopathy is recognized by the same reduced recruitment but with the appearance of denervation potentials at rest (fibrillations and positive sharp waves). As time passes, the motor unit is remodeled, with innervation leading to an increase in MUAP amplitude and duration. This is the hallmark of a chronic lesion. Another uncommon finding linked to chronicity in radiculopathy is chronic repetitive discharge, which is also a form of spontaneous discharge ( Figure 3 ). 9 14

### Severity

There is no consensus on how to graduate severity of radiculopathy. The main approach is to consider the recruitment pattern along with motor NCS.

Greater injury intensities lead to lower recruitment. Recruitment refers to the capability of adding more MUAP as the firing rate increases (contraction force). 9 15 When there are fewer MUAP than expected, recruitment is said to be reduced (with varying degrees), which is associated with neuropathic conditions. 9

As previously stated, the motor conduction studies only alter when there is severe axonal loss. Facing reduction of motor amplitudes, one can infer there is a severe injury of the motor units.

We presented a case of a man with pain radiating from the neck through the left arm for 2 months, with reduced triceps reflex and minor sensory abnormalities. The ENMG scan was consistent with a subacute left C7 radiculopathy, and MRI showed degenerative changes in keeping with this topography. He was treated with nonsteroidal anti-inflammatory drugs for 1 week, along with physical therapy. Pain subsided after 2 months of conservative treatment, and the patient was discharged.

In conclusion, ENMG remains a specific tool for the evaluation of radiculopathy, providing information about localization, temporality, and lesion severity. It also excludes differential diagnosis and better evaluates equivocal or incidental imaging findings. These findings can significantly aid in the clinical decision-making process. We believe that a fundamental understanding of ENMG in the context of CR is essential for training neurologists.
